# Level of miR-101a and miR-107 in Human Adipose Mesenchymal Stem Cells Committed to Insulin-producing Cells

**DOI:** 10.22088/IJMCM.BUMS.10.1.68

**Published:** 2021-05-22

**Authors:** Hadi Rajabi, Somayeh Aslani, Reza Rahbarghazi

**Affiliations:** 1 *Stem Cell Research Center, Tabriz University of Medical Sciences, Tabriz, Iran.*; 2 *Student Research Committee, Tabriz University of Medical Sciences, Tabriz, Iran.*; 3 *Department of Clinical Biochemistry, Faculty of Medicine, Hamedan University of Medical Sciences, Hamedan, Iran.*

**Keywords:** Mesenchymal stem cells, microRNAs, differentiation, insulin-producing cells

## Abstract

Mesenchymal stem cells have the fundamental ability to differentiate into multiple cells such as osteoblasts, neural cells, and insulin-producing cells. MicroRNAs (miRNAs) are single-strand and small non-coding RNAs involved in stem cells orientation into mature cells. There is no comprehensive data about the dynamic of distinct miRNAs during the differentiation of mesenchymal cells from adipose tissue into insulin-producing cells. In this study, we first differentiated adipose-derived mesenchymal stem cells into insulin-producing cells by a three-stepwise protocol. Differentiation capacity was confirmed by the dithizone staining method and hormone (insulin and C peptide) release analysis via electrochemiluminescence technique. In the final phase, the expression of hsa-miR-101a and hsa-miR-107 and two pancreatic genes, sex-determining region Y-box (*SOX*) 6 and neuronal differentiation 1 (*NeuroD1*) were examined during the differentiation procedure on days 0, 7, 14, 21, and 28 after induction, by using real-time PCR assay. The level of C-peptide and insulin were also measured at the end of the experiment. Dithizone staining showed trans-differentiation of adipose-derived mesenchymal stem cells into pancreatic β cells evidenced with red-to-brown appearance compared to the control group, indicating the potency to insulin production. These features were at maximum levels 28 days after cell differentiation. Real-time PCR revealed the increase of *NeuroD1* and reduction of *SOX*6 during differentiation of stem cells toward insulin-producing cells (P <0.05). Both miR-101a and miR-107 showed prominent expression at day 28 (P <0.05). Changes in the expression of miR-101a and miR-107coincided with alteration of *NeuroD1* and *SOX6* that could affect mesenchymal stem cells commitment toward insulin-like beta cells.

MicroRNAs (miRNAs) are endogenous, single-strand, and small non-coding RNAs, miRNAs are regulating mRNAs via targeting their 3^’^-untranslated regions, and contribute therefore to the regulation of protein content (1). Also, studies have revealed prominent regulatory role of miRNAs in the activation of numerous genes participating in progenitor cells differentiation under different circumstances (2). Similar to other stem cell types, miRNAs roles have been investigated regarding β-cells development and islet proliferation (3, 4). The panel of miRNAs profile was previously proven by Wei *et al*. during embryonic stem cells (ESCs) differentiation into insulin-producing cells (IPCs) (5). They revealed that the changes in the expression of miR-375 and miR-7 occurred during ESCs to IPCs differentiation. Mesenchymal stem cells (MSCs) are cells with multipotentiality with the ability to differentiate various cell phenotypes. Commensurate with these descriptions, MSCs are at the center of attention to apply in regenerative medicine (6). MSCs can be obtained from different fetal and adult tissues, however, bone marrow niche and adipose tissue contain an accessible and efficient population of these cells for tissue regeneration aims (7, 8). Easy expansion and collection, and a low content of class I major histocompatibility complex (MHC) molecules make MSCs an ideal choice for regenerative medicine in comparison with other known stem cells (9). To our knowledge, there are gaps in the role of miRNAs expression during the differentiation of MSCS into IPCs. Data analysis revealed the possible effect of miR-101a and miR-107 on target RNAs related to factors such as insulin and sex determining region Y-box 6 (*SOX6*) and neuronal differentiation 1 (*NeuroD1*) (10). In this study, we assessed the possible expression of miR-101a and miR-107 during adipose-derived mesenchymal stem cells (AD-MSCs) differentiation into IPCs.

## Materials and methods

**Cell culture and differentiation**

AD-MSCs line was obtained from Royan Institute (Tehran, Iran). The cells were cultured in low-content glucose Dulbecco’s modified Eagle’s medium (DMEM/LG; Gibco, Germany). To induce cell proliferation, 15% fetal bovine serum (FBS; Gibco, Germany) was added to the culture medium, and plates were transferred to a conventional environment at 37^o^C with 95% humidity and 5% CO_2_. After 80% confluence, cultured cells were detached using trypsin-EDTA (0.25% w/w; Gibco, Germany). AD-MSCs were subjected to the subsequent analyses between passages 3 to 6. 

Three different consequent steps were applied for the differentiation of AD-MSCs to insulin-producing cells as follows: first, cells were pre-induced with DMEM/LG medium supplemented with 5% FBS containing 0.5 mM β-mercaptoethanol (cat no M6250; Sigma-Aldrich, USA) and 10 mM nicotinamide (cat no N0636; Sigma-Aldrich, USA) for 2 days. After completion of the pre-incubation period, the cells were exposed to the high-glucose (4g/L) content DMEM (DMEM/HG; Gibco, Germany) with 2.5% FBS, 0.5 mM β-mercaptoethanol and 10 mM nicotinamide, and were kept for 10 days. Finally, differentiation medium (DMEM/HG) containing 1.5% FBS, 0.5 mM β-mercaptoethanol, 10 mM nicotinamide, and 10 nM exendin-4 (Cat no E7144; Sigma-Aldrich, USA) between passages 3 to 6 was applied for 14 days. The exhausted culture medium was renewed every three days.

**RNA isolation and real time-PCR**

Levels of mir-101a, mir-107, and *NeuroD1* and *SOX*6 were measured during AD-MSCs differentiation to IPC. Both RNAs and miRNAs were extracted on days 0, 7, 14, 21, and 28 by using TRIzol reagent (Cat no T9424; Sigma-Aldrich, USA). The purity and content of RNAs were examined by Nanodrop^®^ (Thermo Scientific, USA). cDNA was synthesized from RNAs by using a cDNA synthesis kit (Exiqon, Vedbaek,Denmark). 50 ng total RNAs were used as template. All primers were designed by Oligo Primer Analysis Software ver. 7 (Table1). The analysis was performed using the BioMolecular Systems (Mo-del: mic) and SYBR Green (Batch no 17D2701; Amplicon, Denmark). MiRNAs expression data were normalized with *U6* RNA and analyzed with the 2^-∆∆Ct^ method. *GAPDH* was used as an internal housekeeping gene for monitoring the expression of *NeuroD1* and *SOX*6.

**Table1:** Sequence of primers

**Genes**	**Primer sequence**	**Tm (°C)**
***GAPDH***	Forward:5’-TGCACCACCAACTGCTTAGC-3’ Reverse:5’- GGCATGGACTGTGGTCATGAG-3’	61
***SOX6***	Forward:5’- CAGCTCTCCACCATGATTACC -3’ Reverse:5’- GTTGTCTCGCAATCTGTTCTTG -3’	58
***NEUROD1***	Forward:5’- CGCTTAGCATCACTAACTGG -3’ Reverse:5’- GGTCATGTTTCGATTTCCTTTG -3’	56
***miR-107***	5’-AGCAGCAUUGUACAGGGCUAUCA-3’	65.4
***miR-101***	5’-UACAGUACUGUGAUAACUGAA-3’	65.4
***U6***	5’-GACTATCATATGCTTACCGT-3’	61.5

**Dithizone staining**

To confirm the differentiation of stem cells toward IPCs, dithizone (DTZ) staining was performed. Briefly, 50 mg DTZ powder (Cat no D5130; Sigma-Aldrich, USA) was dissolved in 5 ml dimethyl sulfoxide (DMSO). To prepare the working solution, the stock solution was diluted to 1:100 in culture medium and then sieved by using a 0.2-μm size strainer. DTZ solution was poured on expanded cells under differentiation medium, and culture plates were kept at 37 °C for 30 min. Following a three time washing with PBS solution and exclusion of background stain, red-colored cells were monitored under an inverted microscope.

**Insulin secretion**

To show the functional activity of IPCs from MSCs source, we measured the levels of specific hormones such as insulin and C peptide after 28 days in the supernatant media by Siemens 06602443 Immulite automated bioanalyzer and Roche insulin and C peptide examination kit, according to manufacturer's instructions. 

**Statistical analysis**

Data are shown as mean ± SD. Statistical analysis was done using One-Way ANOVA and GraphPad Prism version 6.0. P values less than 0.05 were considered as statistically significant.

## Results

**MiR-101a and miR-107 were induced in AD-MSCs differentiating into IPCs**

**Fig. 1:**
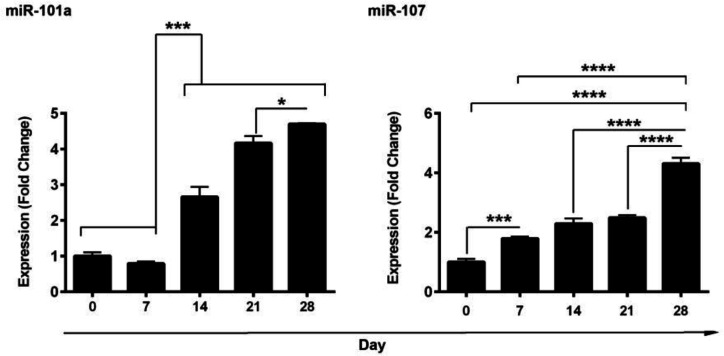
**Evaluation of miR-101a and miR-107 expression over 28 days.** Data showed miR-101a and miR-107 upregulation by time in AD-MSCs exposed to differentiation medium (n=3). One-Way ANOVA and Tukey post-doc analysis. * P < 0.05; *** P < 0.001; **** P < 0.0001

Real-time PCR analysis showed that there was an upward trend in the level of both miR-101a and miR-107 through time, and their levels reached the maximum after 28 days in comparison with the non-treated control cells (Figure 1). Our data noted that miR-101a’s expression did not change in the first 7 days, but its expression was prominently increased after a two-week incubation of AD-MSCs with induction medium. In contrast, the expression of miR-107 was gradually increased and became more evident on day 28 (Figure 1). Despite a slight reduction of miR-101 expression on day 7, the expression of this miRNA tended to increase over time. Therefore, the expressions of miR-101a and miR-107 were at the highest levels on day 28. 

**Dynamics of** ***SOX6*** **and** ***NeuroD1*** **following differentiation of MSCs into IPCs**

**Fig. 2:**
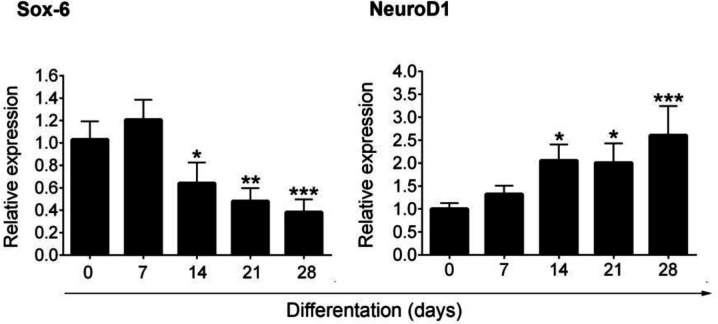
**Real-time PCR analysis of** ***SOX6*** **and** ***NeuroD1*** **genes at different time points**. A reduction in the level *SOX6* coincided with the expression of *NeuroD1*, showing the loss of multipotentiality and differentiation into IPCs (n = 3). One-Way ANOVA and Tukey post-doc analysis.* P < 0.05; *** P < 0.001

Here, we hypothesized that both *SOX*6 and *NeuroD1* genes are tightly controlled by miR-107 and miR-101a, respectively. Both genes participate to the functional maturation of insulin progenitor cells (10,11). To examine the expression of *SOX*6 and *NeuroD1*, a real-Time PCR assay was performed on days 0, 7, 14, 21, and 28. We noted that the expression of *SOX*6 was reduced in progenitor cells subjected to differentiation procedure in comparison with the non-treated control cells (P <0.05). In contrast, *NeuroD1* was found to be upregulated and reached the highest level after 28 days (P<0.05). Both *SOX*6 and *NeuroD1* genes could control the cell destination to IPCs at different stages of cell life (Figure 2). 

**Differentiated IPCs from AD-MSCs was revealed by DTZ staining**

**Fig. 3:**
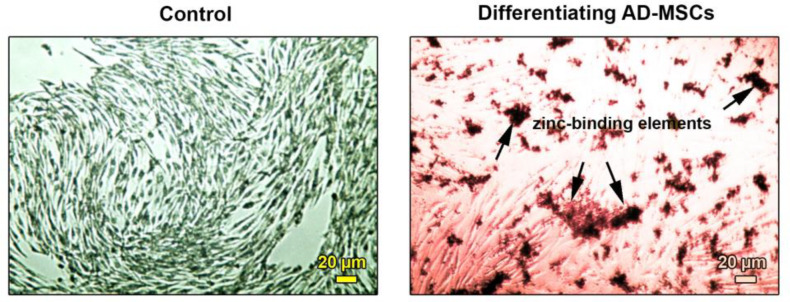
**Measuring AD-MSCs differentiation into IPCs using DTZ staining**. Bright-field imaging of AD-MSCs at passage three: the cells show a fibroblast-like appearance and spindle shape (left). Determination of AD-MSCs differentiation into IPCs after 28 days and upon staining with DTZ (right). Imaging revealed the existence of red-color zinc-binding elements in beta cells (arrows) showing intracellular accumulation of Zn, and indicating insulin-like cell activity

Red-colored DTZ positive cells were found after 28 days in cells incubated with differentiation medium in comparison with the control group (Figure 3). Commensurate with these descriptions, insulin and zinc components were produced in differentiated cells.

**Fig. 4:**
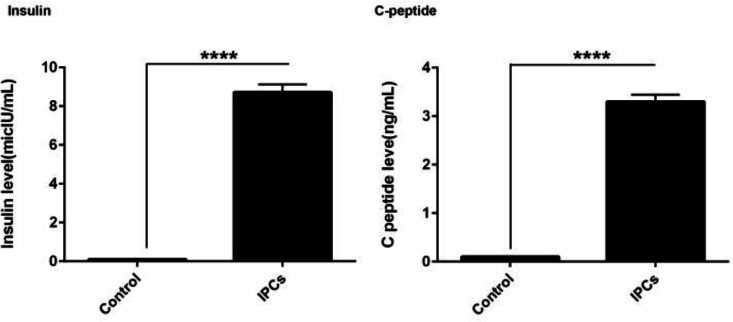
**Detection of Insulin and C-peptide in the supernatant of Ad-MSCs orientated toward IPCs after 28 days.** Data showed a significant increase in the level of both insulin and C-peptide in comparison with the non-treated control AD-MSCs (n = 3). Student t-test; **** P < 0.0001

**Insulin and C peptide were secreted from IPCs**

A significant increase in insulin (P = 0.0011) and C peptide (P = 0.001) levels was observed in the supernatant of the treatment group in comparison with the control group (Figure 4). These results showed that our protocol was eligible to induce the paracrine activity of IPCs by releasing insulin and C peptide.

## Discussion

Stem cells are known as an interesting cell source for alleviating various pathological conditions like neurodegenerative (12), cardiovascular (13), and pancreatic disorders (14), and allergic pulmonary diseases like asthma (15). In addition to easy cultivation and expansion of MSCs, these cells can preserve differentiation potential under long culture conditions (16). Therefore, MSCs are a more efficient and well-choice option to accelerate the regeneration of injured tissues. 

Previous studies have confirmed MSCs potential to generate IPCs. Knowing the underlying molecular mechanism in the regulation of stem cell fate and orientation helps us to obtain better therapeutic effects. MiRNAs are one of those regulatory elements involved in the regulation of various bioactivities (17). Interruption of miRNAs biogenesis could lead to acute disorders. For instance, it has been shown that the malfunction of Dicer 1, an enzyme involved in miRNAs synthesis, can regulate the islet structure and function of β-cells in response to systemic glucose levels (18). There are a few experiments related to the dynamic of miRNAs in IPCs originated from different stem cells source. Wei *et al*. have detected the expression of miR-375, -7, -146a, and -34a in embryonic stem cells directed to IPCs. They also reported a diverse expression pattern in these cells, indicating the specific role of each miRNA in the dynamic of stem cells toward IPCs (5). Sebastiani *et al*. monitored the transcription of eighteen miRNAs, and found that the activity of these miRNAs is integral to stem cell differentiation toward IPCs. Based on their experiments, the transcription of other miRNAs such as miR-135a, miR-138, miR-149, and miR-375 coincided with the suppression of miR-31, miR-127, miR-143, miR-373 (19).

The effective roles of miR-101a and miR-107 have been investigated in different biological processes (20, 21). However, their expression during stem cell differentiation to IPCs has not been studied yet. In this study, we aimed to find changes in the expression of miR-101a and miR-107 during AD-MSCs differentiation into IPCs (10, 11). According to our study, the expression of both miR-101a and miR-107 was induced, and coincided with the modulation of *SOX*6 and *NeuroD1* genes. The current study showed that *SOX6* was downregulated, indicating the stemness removal while *NeuroD1* was upregulated and reached a peak level on day 28 of differentiation. These data suggest that SOX6 could be considered as a unique cell marker for IPCs differentiated from AD-MSCs. These features show the phenotype acquisition of IPCs in studied cells subjected to our protocol. There are multiple limitations regarding the current experiment. We suggest future studies focusing on expression analysis of multiple miRNAs simultaneously and during a long period. Protein levels of different factors should also be monitored for more accurate evaluation of MSCs differentiation potential. Performing prolonged *in vitro* experiments could help to monitor the expression of these miRNAs over time. Investigating the relationship between different miRNAs and pancreatic specific factors may also help to a better understanding of differentiation mechanisms.
